# Intraosseous bone marrow concentrate delays total hip arthroplasty in osteoarthritis: A fifteen year matched cohort study with dose–response analysis

**DOI:** 10.1007/s00264-026-06908-x

**Published:** 2026-06-16

**Authors:** Philippe Hernigou, Christopher J. Centeno, Dustin R. Berger, Ehren Dodson, Matthew B. Murphy

**Affiliations:** 1 Paris-Est Créteil University, Créteil, France; 2 Hôpitaux Universitaires Henri-Mondor, Créteil, France; 3 Centeno-Schultz Clinic, Broomfield, United States; 4 Regenexx, Broomfield, United States; 5 Regenexx, Broomfield, United States

**Keywords:** Cell therapy, Hip osteoarthritis, Total hip arthroplasty, Bone marrow concentrate, Colony forming unit-fibroblast, Revision surgery

## Abstract

**Purpose:**

To evaluate whether intraosseous (IO) autologous bone marrow concentrate (BMC), containing mesenchymal stem cells (MSCs) enumerated in vitro as colony forming unit-fibroblasts (CFU‑F), reduces or delays conversion to total hip arthroplasty (THA) compared with matched conservative care, and to assess the influence of disease progression and CFU‑F dose on outcomes.

**Methods:**

A monocentric matched cohort (n = 434 hips; 217 BMC, 217 control) was followed for up to 15 years. Bone marrow was aspirated from the iliac crest, processed into BMC to concentrate nucleated cells, and injected intraosseously into the femoral head under fluoroscopy. Demographics, BMI, osteoarthritis grade, and CFU‑F were recorded. The primary outcome was THA‑free survival by Kaplan–Meier with log‑rank testing. Cox proportional-hazard models provided adjusted effects and predictions.

**Results:**

Progression to THA after 15 years occurred in 16.1% (35 of 217) of patients receiving IO BMC versus 40.1% (87 of 217) of control patients receiving conservative care. Kaplan–Meier curves demonstrate superior THA‑free survival with BMC overall, mild osteoarthritis, and with increased CFU‑F dose. No adverse events were observed. Revision surgeries were not performed in those receiving BMC (0 of 35), compared to an 8.0% revision rate among control patients (7 of 87).

**Conclusion:**

IO BMC was associated with substantially delayed progression to THA with a CFU‑F dose–response and favourable safety profile. These findings suggest that IO BMC may represent a clinically meaningful joint-preserving option for selected patients with hip osteoarthritis, particularly when performed before advanced disease progression and with higher CFU-F doses.

## Introduction

Hip osteoarthritis (OA) imposes a substantial symptomatic and functional burden [1]. Following unilateral THA for OA, the contralateral hip frequently deteriorates over time with significant risks of subsequent arthroplasty. Up to 78% of THA patients show radiographic evidence of OA in the contralateral hip, with contralateral THA occurring in 22–53.8% of patients [2–5]. High rates of bilateral or ipsilateral radiographic OA and structural risk factors such as dysplasia, cam morphology, and femoroacetabular impingement further contextualize progression risk [6–12].

Biologic, joint preserving strategies, such as intraosseous (IO) autologous bone marrow concentrate (BMC), have shown promising disease modifying effects. A clinically important population of cells found in BMC are the mesenchymal stem/stromal cells (MSC). Enumerated as colony-forming unit-fibroblasts (CFU-F), MSCs possess the ability for self-renewal and can be differentiated in vitro into multiple connective tissue cell types, including osteoblasts and chondrocytes [13]. With respect to the treatment of OA, MSCs are thought to modulate the joint microenvironment, having effects on inflammation, extracellular matrix turnover, chondrocyte function, and alleviation of pain [14]. Because OA pain is multifactorial and often arises from richly innervated joint tissues such as the subchondral bone rather than from aneural cartilage itself, the anti-inflammatory and potentially neuromodulatory effects of delivered MSCs may contribute directly and indirectly to pain relief [14–16].

Considering the challenge of conducting randomized trials in patients with painful hips, we have implemented a study design using the contralateral hip of patients undergoing THA as an internal control. During the same anesthetic session as their primary THA, patients received a subchondral IO injection of BMC in the non-operated contralateral hip as a first-line treatment, potentially delaying the need for a second arthroplasty. Our hypothesis was that direct subchondral IO BMC injection could also provide symptom relief by supporting the bone structure through cell-mediated repair. We evaluated whether intraosseous BMC reduces or delays conversion to THA versus matched conservative care and examined predictors including CFU-F dose and radiographic progression of OA.

## Materials and methods

### Study design and participants

A monocentric matched cohort study under ethics approval and the Declaration of Helsinki was conducted in accordance with STROBE guidelines [17]. We identified 217 adults who underwent a contralateral IO BMC injection at the time of hip arthroplasty between 2000–2010 with a minimum of 15 years follow-up. As a natural-history control, 217 patients who underwent unilateral hip arthroplasty with conservative care on contralateral hip were matched by OA etiology, Kellgren-Lawrence (KL) grade, age (± 10 years), and sex.

### Procedures

All procedures were performed under anesthetic during a single event by the same surgical team. The procedural sequence included a bone marrow aspiration from the iliac crest using small volume aliquots from multiple perforations (three to five) through a single skin entry point to limit peripheral‑blood dilution. Approximately 50 mL of BMA was aspirated from both posterior iliac crests (100 mL total) with a ten mL syringe, using rapid pulls of approximately three mL as described to maximize CFU-F recovery [18, 19]. All instruments were rinsed with a heparin solution prior to aspiration, while the BMA was mixed with 10% ACD-A upon collection for anticoagulation. The BMA was subsequently centrifuged to capture the buffy coat as BMC. The hip with greater pain underwent a THA via posterolateral approach with a uniform implant (Ceraver Osteal, Roissy, France), including an anodized titanium alloy (TiAl6V4) stem that was smooth and cemented. The ceramic head measured 32 mm in diameter and was secured with a Morse taper. Patients within the IO BMC cohort received a contralateral intraosseous BMC injection (≈20 mL) through a four mm trephine into the femoral head under biplanar fluoroscopy. Additional ilium injections were performed when indicated (acetabular cysts). Peri‑procedural anti‑inflammatories and analgesics were withheld three to four weeks prior to BMC treatment. Postoperative care included approximately ten days of protected weight‑bearing. No formal physical therapy was required for the IO BMC‑treated hip. Conservative management of hip OA for those in the control cohort focused on relieving pain, improving joint function, and slowing disease progression without surgery. It typically involved patient education, weight management, physical therapy, and exercise programs to strengthen muscles and maintain mobility, as well as pain control with analgesics or non-steroidal anti-inflammatory drugs. Assistive devices (e.g., a crutch) and lifestyle modifications were recommended where necessary.

### BMC dose quantification and outcome metrics

The total number of CFU‑F measured in vitro was used as a surrogate for MSC dose and was determined as the product of nucleated cell concentration, CFU-F frequency, and injection volume. The primary endpoint was time to contralateral THA (THA‑free survival). Secondary outcomes included revision THA, pain trajectories, medication use, and radiographic progression. Routine follow-up was carried out every year following the initial THA with patients completing standardized outcome questionnaires including a contralateral hip assessment. Telephone follow-up was conducted if a clinical visit was missed. Pain in the contralateral hip was assessed with the pain component of the Harris hip score and graded as none, slight, mild, moderate, severe, or disabling. The time to the development (or changing of intensity) of contralateral hip pain relative to baseline scores was recorded as was patient use of pain medication (opioid, non-steroidal anti-inflammatory drugs, acetaminophen, muscle relaxant).

Radiographic evaluation was performed prior to treatment and during post-treatment follow-up visits. Radiographs were obtained using standardized protocols and included standing anteroposterior pelvic, and false-profile views. Two trained researchers made bilateral hip measurements of KL osteoarthritis grade preoperatively and at the most recent patient follow-up evaluated. The following radiological criteria were used: KL1—possible osteophytic lipping with uncertain joint space narrowing; KL2—presence of definite osteophytes with potential joint space narrowing; KL3—moderate multiple osteophytes, evident joint space narrowing, some sclerosis, and possible bone-end deformity; KL4—large osteophytes, significant joint space narrowing, severe sclerosis, and clear bone-end deformity. Severity of hip OA was classified as mild (KL1/2), moderate (KL3), or severe (KL4).

### Statistical analysis

THA-free survival was calculated from the date of initial treatment to the date of conversion to contralateral THA, which was the primary endpoint. Initial treatment was defined as the unilateral THA procedure, during which patients either received contralateral IO BMC or proceeded with conservative management of the contralateral hip. Patients who did not undergo contralateral THA were censored at the last documented follow-up, with follow-up truncated at 15 years. Kaplan–Meier methods were used to estimate THA-free survival and curves were compared using log-rank tests. Survival analyses were performed for the overall IO BMC and control cohorts and were repeated after stratification by baseline OA severity. Cumulative THA conversion at 5, 10, and 15 years was defined as conversion occurring at or before the specified follow-up time point and was reported as event counts and percentages.

Cox proportional-hazards models were used to evaluate factors associated with conversion to THA [20, 21]. All included hips had complete data for the variables used. The proportional-hazards assumption was assessed using scaled Schoenfeld residual plots and time-trend tests. Candidate predictors included study cohort, age, BMI, and baseline OA severity. Age and BMI were analyzed as continuous variables and are reported as hazard ratios per ten year and 5 kg/m^2^ increments, respectively. OA severity was modeled as an ordered clinical severity variable from mild to severe. Because CFU-F dose was applicable only to patients treated with IO BMC, dose–response analyses were performed within the IO BMC cohort. CFU-F dose was modeled as a continuous predictor and reported as hazard ratios per 10,000 CFU-F, while CFU-F tertiles were used for descriptive Kaplan–Meier and cumulative conversion summaries.

To characterize the relationship between transplanted cell dose and THA-free survival, CFU-F count was analyzed as a continuous variable after transformation using ln(1 + CFU-F count/100,000) to reduce skewness and to model diminishing incremental effects at higher cell doses. A ridge-penalized Cox proportional-hazards model was used to improve model stability given the number of events and the inclusion of severity-specific dose effects. The model included transformed CFU-F dose, baseline OA severity, and interaction terms between CFU-F dose and OA severity, allowing the association between dose and THA-free survival to vary by disease severity. Predicted THA-free survival was then estimated for each OA severity level using the corresponding untreated control survival at five, ten and 15 years as the natural-history anchor. All analyses were performed using two-sided tests, and p < 0.05 was considered statistically significant.

## Results

The average patient age upon initial THA was 42.2 years (ranging from 18 to 60) and patient BMI was 25.6 kg/m^2^ (ranging from 19.3 to 32.9). No differences were observed between the BMC and matched control cohorts with respect to age, gender, OA grade and distribution of developmental diseases contributing to secondary OA apart from BMI, which was higher in the control cohort (25.2 vs. 26.0) Table 1. The average total CFU-F count from those receiving BMC (n = 217) was ~ 104 × 10^3^ (ranging from 55 × 10^3^ to 150 × 10^3^). Progression to contralateral THA occurred in 16.1% (35 of 217) of patients receiving BMC versus 40.1% (87 of 217) of control patients receiving conservative care. Surgical revisions were performed in zero of 35 BMC patients versus seven of 87 control patients, respectively. Cumulative THA rates were 4.6% versus 28.6% at five years, 12.0% versus 35.9% at ten years in the BMC and control cohorts, respectively.

**Table 1:** Demographics of patients comprising the IO BMC and control cohorts. Kellgren-Lawrence (KL), Developmental Dysplasia of the Hip (DDH); Legg-Calvé-Perthes (LCP); Slipped Capital Femoral Epiphysis (SCFE); Coxa Profunda (CP); *Significantly different by unpaired t-test

		IO BMC Cohort	Control Cohort
Patients (Females)		217 (116)	217 (116)
Age (Years)		41.8 ± 9.0	42.7 ± 11.6
BMI (kg/m^2^)		25.2 ± 3.1*	26.0 ± 3.3*
OA Grade	KL1	13	13
KL2		95	95
KL3		72	72
KL4		37	37
Primary OA		115	115
Secondary OA		102	102
DDH		38	33
LCP		34	36
SCFE		13	14
CP		17	19

Patient age and BMI were not associated with THA conversion. The hazard ratio (HR) per ten years was 1.04 (95% CI 0.88 to 1.22, p = 0.65) and the HR per 5 kg/m^2^ was 1.01 (95% CI 0.76 to 1.33, p = 0.96). However, study cohort and OA severity were associated with THA conversion. The HR for study cohort was 3.45 (95% CI 2.33 to 5.21, p < 0.001) and the HR for OA severity was 2.35 (95% CI 1.90 to 2.92, p < 0.001). Similarly, when analyzing only the IO BMC cohort, patient age and BMI were not associated with THA conversion, while CFU-F dose and OA severity were. The HR per ten years for the IO BMC cohort was 0.77 (95% CI 0.52 to 1.13, p = 0.18) and the HR per 5 kg/m^2^ was 0.96 (95% CI 0.51 to 1.80, p = 0.89), whereas the HR per 10 K CFU-F was 0.51 (95% CI 0.41 to 0.63, p < 0.001) and the HR for OA severity was 2.82 (95% CI 1.84 to 4.48, p < 0.001). Evidence of time-dependence was observed for study cohort in the combined cohort model and CFU-F dose in the IO BMC cohort model. Accordingly, model estimates should be interpreted as summary hazard ratios over follow-up rather than time-invariant effects.

The THA conversion rates for IO BMC cohort patients aged < 40 years, 40–50 years, and > 50 years were 12% (11 of 90), 27% (24 of 88), and 0% (0 of 39), respectively. Developmental dysplasia of the hip (DDH) was associated with higher THA conversion rates compared to borderline or non-dysplastic hips and was more common in younger patients. In the IO BMC cohort, among the 38 hips with DDH, ten required THA over the 15-year follow-up period, corresponding to a conversion rate of approximately 26.3%. In contrast, among the 179 non-dysplastic hips, 25 required THA, corresponding to a lower conversion rate of approximately 14.0%.

**Fig. 1:**
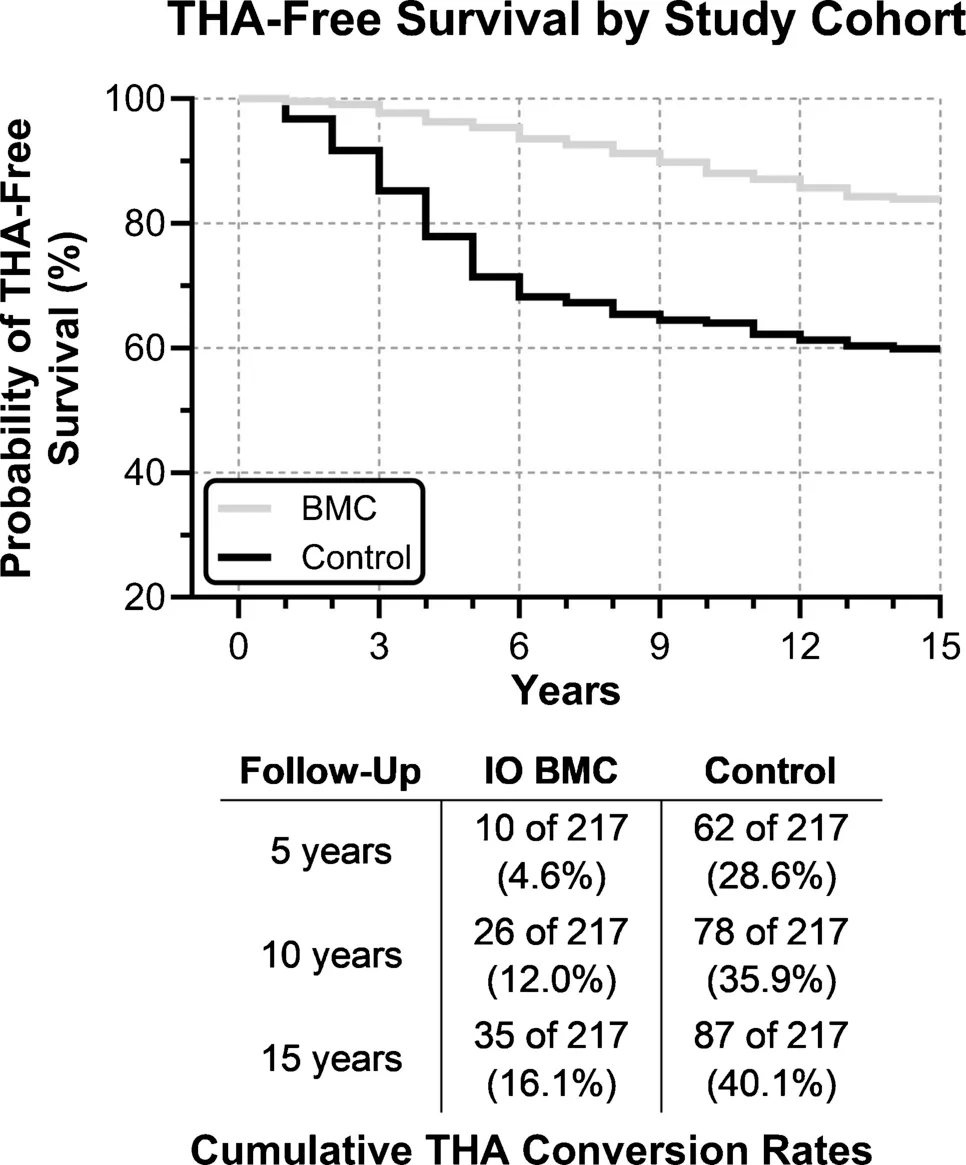
Kaplan–Meier curves comparing THA‑free survival for IO BMC versus control cohorts over 15 years. Curves are significantly different by log-rank test, p < 0.001

**Fig. 2:**
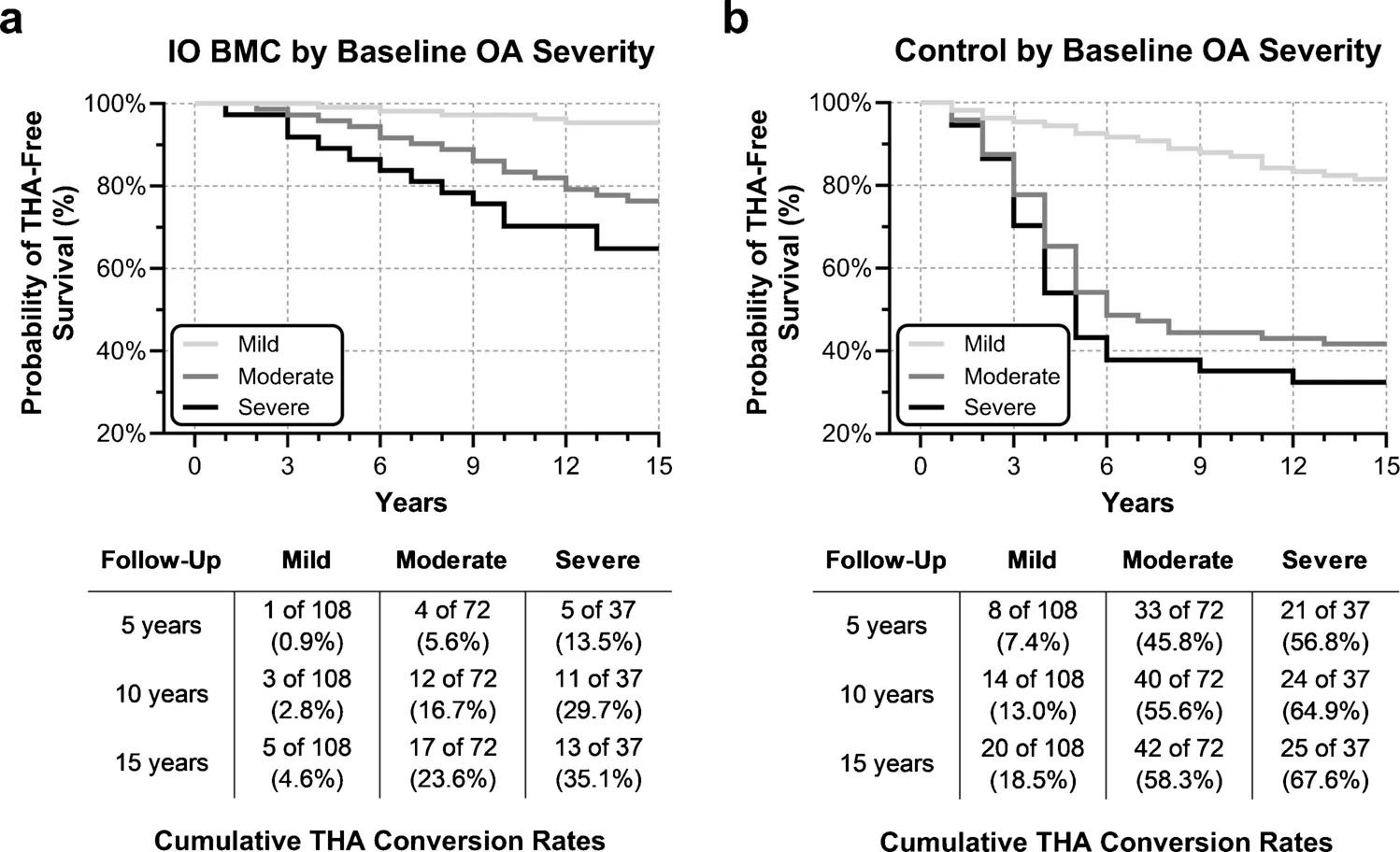
IO BMC cohort (**a**) and control cohort (**b**) Kaplan–Meier curves comparing THA-free survival stratified by OA progression (Mild = KL1/2, Moderate = KL3, and Severe = KL4) over 15 years. Curves are significantly different by log-rank tests, p < 0.001

Kaplan–Meier curves show superior THA‑free survival for BMC overall (Fig. 1). When analyzed across OA severity, both BMC and control cohorts show superior THA-free survival among those with mild OA compared to more advanced disease progression (Fig. 2a-b). At the final 15-year follow-up, THA surgery rates by baseline OA severity were 4.6% (5 of 108) for mild, 23.6% (17 of 72) for moderate, and 35.1% (13 of 37) for severe OA among those in the IO BMC cohort. In contrast, THA surgery rates in the control cohort were 18.5% (20 of 108) for mild, 58.3% (42 of 72) for moderate, and 67.6% (25 of 37) for severe OA.

**Fig. 3:**
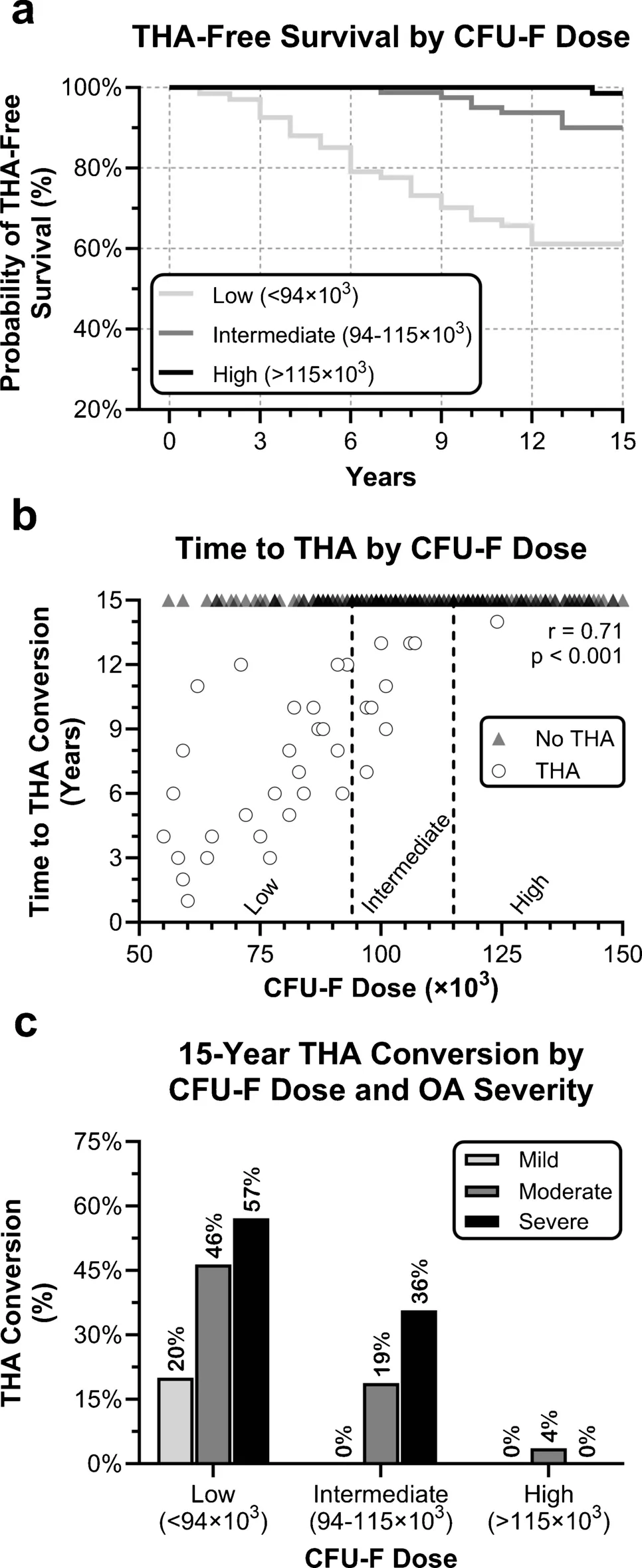
Dose–Response relationship between transplanted CFU-F dose and THA-free survival in the IO BMC cohort. Kaplan–Meier curves stratified by CFU-F dose; low (< 94 × 10^3^), intermediate (94 × 10^3^ to 115 × 10^3^), high (> 115 × 10.^3^) over 15 years. Curves are significantly different by log-rank test, p < 0.001 (**a**). Scatterplot of CFU‑F dose versus time to THA conversion. Circles denote hips converted to THA, and triangles denote THA-free survival (censored observations at 15 years). The vertical reference lines mark the low, intermediate and high CFU-F dose tertiles. A significant relationship was observed, Spearman coefficient = 0.71 and p < 0.001 (**b**). Rate of THA conversion at 15 years (%) by CFU‑F dose and baseline OA severity (**c**)

**Fig. 4:**
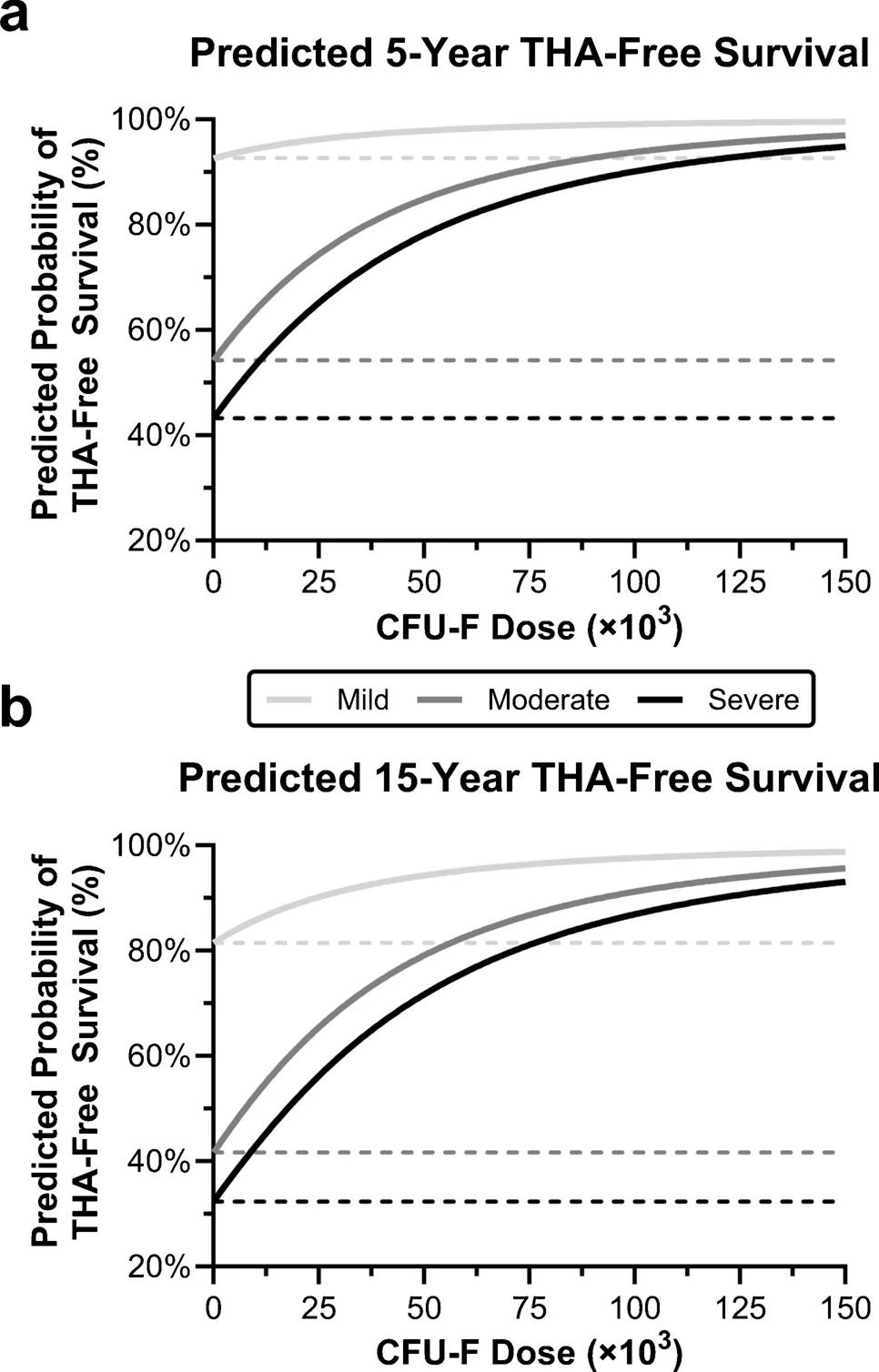
Predicted THA-free survival at five years (**a**) and 15 years (**b**) by transplanted CFU-F dose and OA severity (Mild = KL1/2, Moderate = KL3, and Severe = KL4). Each curve is anchored to the observed Kaplan–Meier survival of the untreated control cohort for the corresponding OA grade (dashed horizontal lines), illustrating incremental improvement in joint preservation predicted with higher MSC doses

Upon dividing the IO BMC cohort into thirds based on CFU-F dose, patients in the upper two-thirds of CFU-F dose demonstrated no THA conversion for at least six years (Fig. 3a). Cumulative THA surgery rates were 38.8% (26 of 67), 10.0% (8 of 80), and 1.4% (1 of 70) in those treated with less than 94 × 10^3^, 94 × 10^3^ to 115 × 10^3^, and more than 115 × 10^3^ CFU-F, respectively. All survival analyses resulted in significantly different Kaplan–Meier curves by log-rank tests (p < 0.001). A scatterplot of CFU‑F dose versus years until THA demonstrates that higher CFU‑F counts are associated with longer THA‑free intervals, with many high‑dose cases censored at 15 years and earlier conversions clustered at lower doses (Fig. 3b). A categorical summary of THA conversion percentages by CFU‑F dose across OA severity at baseline highlights higher conversion rates at lower doses and more advanced disease progression (Fig. 3c). To explore the relationship between CFU-F dose and long-term hip survival, we constructed predictive survival models anchored to the natural history of untreated patients (Fig. 4a-b).

At the terminal assessment (15 years or at the time of contralateral THA), the distribution of KL grades favored IO BMC compared to conservative care in terms of radiographic progression of OA. In the IO BMC cohort, 33.2% of hips were classified as mild OA (KL1 4.6%, KL2 28.6%) versus 23.9% (KL1 3.2%, KL2 20.7%) in the control cohort. Severe OA (KL4) accounted for 29.0% of hips in the IO BMC cohort versus 33.6% in controls, while moderate OA (KL3) accounted for 37.8% versus 42.4%, respectively. Relative to the initial combined distribution (KL1 6.0%, KL2 43.8%, KL3 33.2%, KL4 17.1%), both cohorts progressed toward higher KL grades over time, but the IO BMC cohort maintained a smaller share of advanced OA grades (KL3/4 66.8% BMC versus 76.0% control), consistent with delayed radiographic progression.

The mean Harris hip score improved from 76 points (ranging from 65 to 82) prior to treatment to 90 points (ranging from 85 to 96) at the terminal assessment, resulting in a 14-point gain among the 182 IO BMC patients without THA conversion. Pain was the most important contributor to improvement, increasing from 26 to 40 points. In contrast, the mean Harris hip score decreased from 77 points (ranging from 63 to 84) to 71 points (ranging from 64 to 81) over the same period, resulting in a 6-point loss among the 130 control patients without THA conversion. Pain was the most important contributor to deterioration, decreasing from 28 to 23 points.

Most patients reported using pain medication prior to treatment with 52.1% (113 of 217) and 56.2% (122 of 217) of patients taking opioids, non-steroidal anti-inflammatory drugs, acetaminophen, and/or muscle relaxants in the IO BMC and control cohorts, respectively. However, at the terminal assessment, only 19.2% (35 of 182) of patients in the IO BMC cohort without THA conversion reported use of pain medication, whereas use of pain medication was reported by all 130 control patients without THA conversion.

## Discussion

Treatment with IO BMC into the subchondral bone was associated with substantially lower THA conversion and fewer subsequent revision surgeries compared with matched natural history. Across all initial OA grades (KL1-KL4), the IO BMC cohort showed slower radiographic worsening than the control cohort, with more hips remaining as mild OA and fewer progressing to severe OA at the terminal assessment. The CFU‑F dose–response supports biological plausibility and aligns with external literature reporting symptomatic benefit and acceptable safety of autologous bone marrow-derived products for hip OA [22–26] as well as multicenter safety surveillance of orthopaedic autologous cell procedures [27]. Our predictive models suggest that while modest CFU-F doses may be sufficient to maintain long-term hip survival in mild OA, substantially higher doses are likely required to approach durable joint preservation in more advanced OA.

Importantly, CFU-F dose depends on aspiration strategy and injectate allocation. Using smaller aspiration volumes with small syringes enrich CFU‑F by minimizing peripheral‑blood dilution [18]. Additionally, the realized CFU‑F dose depends not only on marrow aspiration technique but also on the concentration method and the manner of intraosseous injection, so that the number of viable CFU‑F delivered to subchondral bone is maximized. A prior study by Centeno et al. comparing intra‑articular (IA) BMC with combined intraosseous plus intra‑articular (IO + IA) BMC observed no significant differences in outcomes [28]. However, their protocol used modest bone marrow aspiration volumes and allocated only a small fraction of the prepared BMC intraosseously, likely reducing the CFU‑F dose delivered into the bone and potentially attenuating IO‑specific effects.

The earliest IO bone marrow-based procedures in the hip were developed for the treatment of osteonecrosis of the femoral head, with the primary aim of preserving the femoral head and preventing subchondral collapse beneath the articular cartilage. Disruption of the femoral head blood supply can lead to osteocyte death and structural weakening of the necrotic subchondral bone. Because the intrinsic reparative response is insufficient, disease progression often culminates in structural failure of the necrotic bone and collapse of the femoral head if untreated. Early intervention with procedures such as core decompression augmented with autologous BMC has therefore been explored to enhance bone repair and delay or prevent collapse [29–31]. Although the pathological process of OA differs from that of osteonecrosis, OA is now understood as a whole-joint disease characterized not only by cartilage degeneration but also by abnormal remodeling of the subchondral bone, bone marrow lesions, synovial inflammation, and altered osteochondral crosstalk [32, 33]. Structural deterioration in OA typically progresses more gradually than in osteonecrosis and involves progressive cartilage wear, changes in subchondral bone architecture, and, in some cases, focal areas of bone damage or insufficiency that contribute to deformation of the femoral head and joint incongruity over time [32].

Because subchondral bone plays an active role in OA pathophysiology, biologic treatments targeting this compartment have been proposed. Delivery of IO BMC into the subchondral bone has been investigated as a strategy to modify the local microenvironment by providing MSCs and other marrow-derived elements capable of modulating inflammation, supporting tissue repair, and influencing bone remodeling. Preliminary clinical studies and case series have suggested that subchondral BMC injections may improve symptoms and potentially influence disease progression, although larger controlled trials are still needed to confirm structural effects in hip OA [34–36]. The biological rationale for this approach is supported by evidence from marrow-stimulation and cartilage repair research showing that access to the bone marrow compartment can stimulate the formation of reparative fibrocartilaginous tissue at the osteochondral junction. Histologically, marrow-derived repair tissue has been described as fibrocartilage arising from marrow spaces in the subchondral bone and extending toward the articular surface, often forming small projections from the bone-cartilage interface toward the joint surface [37, 38]. One potential mechanism by which IO BMC may delay progression toward THA is through direct targeting of abnormal subchondral bone remodeling. In OA, the subchondral compartment undergoes sclerosis, trabecular microdamage, cyst formation, altered vascularity, and increased bone turnover. Osteoarthritis is increasingly recognized as a disease of the whole joint rather than an isolated cartilage disorder, with important interactions among cartilage, subchondral bone, bone marrow lesions, synovium, and inflammatory mediators [15, 39]. These changes may precede visible cartilage degeneration and contribute to pain generation through richly innervated bone tissues. By delivering concentrated marrow-derived cells directly into the femoral head, IO BMC may act at the level where structural failure and inflammatory signaling originate.

Mesenchymal stem/stromal cells, quantified in this study as CFU-F, are thought to exert their effects primarily through paracrine signaling rather than direct tissue replacement. MSCs secrete a broad range of anti-inflammatory cytokines, growth factors, extracellular vesicles, and immunomodulatory mediators capable of modifying the osteoarthritic microenvironment [14–16]. These mediators may suppress pro-inflammatory pathways involving interleukin-1β, tumor necrosis factor-α, and matrix metalloproteinases, thereby reducing catabolic signaling within both cartilage and subchondral bone. At the same time, MSC-derived factors may stimulate local repair processes, angiogenesis, extracellular matrix synthesis, and regulation of osteoblast and osteoclast activity.

The subchondral localization of the injection may be particularly important. Intra-articular injections primarily expose synovial tissues and cartilage surfaces to biologic products, whereas intraosseous delivery targets the bone-cartilage interface directly [35]. The osteochondral junction represents an active biological interface where biochemical and mechanical signals are exchanged between cartilage and bone and allow the production of new fibrocartilage [37, 38]. Abnormal crosstalk across this interface contributes to OA progression. IO BMC may therefore influence disease progression by restoring a more physiological subchondral environment and reducing pathological remodeling beneath the cartilage surface.

**Fig. 5:**
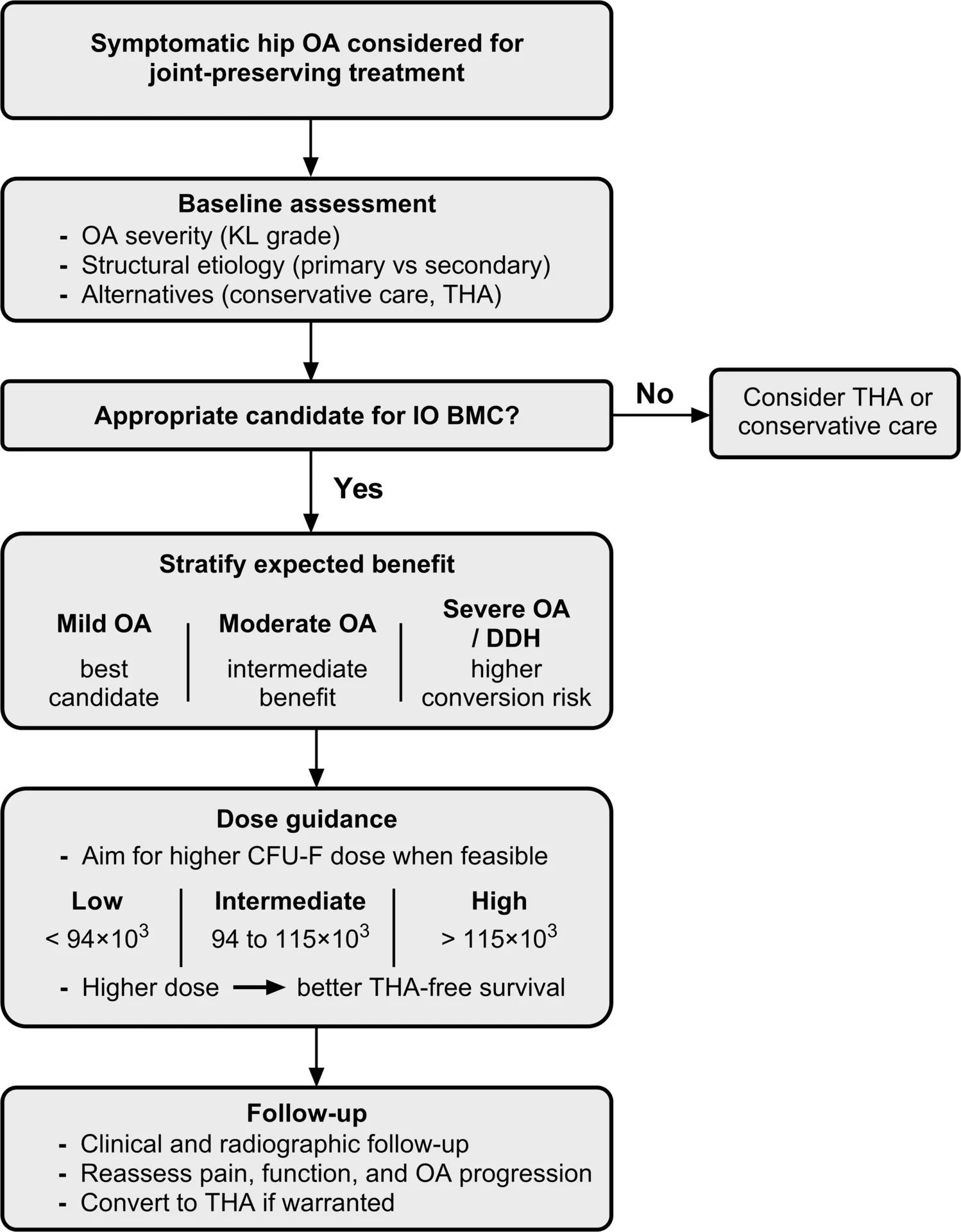
Clinical decision algorithm. Patients with symptomatic hip osteoarthritis should first undergo clinical and radiographic staging. Intraosseous BMC appears most appropriate in mild-to-moderate disease, particularly when an adequate CFU-F dose can be delivered. Advanced OA, structural deformity such as DDH, and low CFU-F dose are associated with a higher risk of conversion to THA and should be discussed during shared decision-making

Our findings support CFU‑F dose‑response and minimum effective dose observations across orthopaedic indications, including bone nonunion, ankle and shoulder osteonecrosis, knee OA, and intradiscal applications [40–44]. However, patient selection appears important as primary OA with mild disease progression showed the strongest benefit in our cohort, whereas developmental etiologies conveyed higher conversion risks, even with BMC. Hips with DDH had nearly twice the likelihood of requiring surgical intervention compared with those without dysplasia. These findings support the concept that underlying structural abnormalities, such as DDH, are associated with less favorable long-term outcomes, even when treated with bone marrow-based therapies. Therefore, we propose a clinical decision algorithm for intraosseous BMC treatment of hip osteoarthritis (Fig. 5).

Several limitations should be acknowledged. This was a retrospective, non-randomized, single-centre matched cohort study, and therefore causality cannot be established. Although matching was performed by OA aetiology, KL grade, age and sex, residual confounding and selection bias may remain. Patients who elected IO BMC may have differed from those managed conservatively in ways that were not fully captured, including symptom severity, expectations, activity level, willingness to pursue joint-preserving therapy, or thresholds for later THA conversion. The potential for placebo effects due to the absence of a sham injection group should be considered. However, the observed CFU-F dose–response relationship in the present study supports a biological effect rather than a placebo phenomenon. Higher CFU-F counts were associated with prolonged THA-free survival, particularly in mild and moderate OA, suggesting that the concentration of progenitor cells and their associated trophic activity may be critical determinants of long-term efficacy. Yet, the single-center design and use of a highly standardized technique performed by an experienced team may also limit generalizability to other clinical settings.

Radiographic measurements may have failed to detect structural abnormalities, such as femoral version, acetabular version, or subtle dysplasia, that could influence symptoms and disease progression. Although control hips were matched by OA severity using KL grade, radiographic matching alone may not fully account for differences in joint mechanics or structural risk factors. Additionally, the mechanisms underlying DDH progression remain unclear, and it is uncertain whether changes in joint mechanics may limit the response to bone marrow-based therapy. Despite these limitations, the sustained differences in THA-free survival, radiographic progression, pain levels, and medication use support the hypothesis that IO BMC may have clinically meaningful joint-preserving effects. These findings should be interpreted as supportive rather than definitive and warrant prospective randomized, sham-controlled, multi-centre validation.

By reducing pain, delaying disease progression, and preventing the need for THA, IO BMC therapy represents a valuable non-surgical alternative that could shift the treatment paradigm for early-to-moderate OA. Given its demonstrated efficacy, IO BMC therapy could be considered as a first-line intervention for patients, especially those seeking to preserve native joint function and delay arthroplasty.

## Data Availability

All data supporting the findings of this study are available from the corresponding author upon reasonable request.
